# The World Trade Center Exposome and Health Effects in 9/11 Rescue and Recovery Workers

**DOI:** 10.21203/rs.3.rs-3482965/v1

**Published:** 2023-12-12

**Authors:** Elza Rechtman, Michelle Rodriguez, Elena Colicino, Christopher Hahn, Esmeralda Navarro, Azzurra Invernizzi, Christopher Dasaro, Susan Teitelbaum, Andrew Todd, Megan Horton

**Affiliations:** Icahn School of Medicine at Mount Sinai, New York, NY; Icahn School of Medicine at Mount Sinai, New York, NY; Icahn School of Medicine at Mount Sinai; Icahn School of Medicine at Mount Sinai, New York, NY; Icahn School of Medicine at Mount Sinai, New York, NY; Department of Environmental Medicine and Public Health, Icahn School of Medicine at Mount Sinai; Icahn School of Medicine at Mount Sinai, New York, NY; Icahn School of Medicine at Mount Sinai, New York, NY; Icahn School of Medicine at Mount Sinai, New York, NY; Department of Environmental Medicine and Public Health, Icahn School of Medicine at Mount Sinai

## Abstract

In the aftermath of the World Trade Center (WTC) attack, rescue and recovery workers faced hazardous conditions and toxic agents. Prior research linked these exposures to adverse health effects, but mainly examined individual factors, overlooking complex mixture effects. This study applies an exposomic approach encompassing the totality of responders’ experience, defined as the WTC exposome. We analyzed data from 34,096 members of the WTC Health Program General Responder, including mental and physical health, occupational history, traumatic and environmental exposures using generalized weighted quantile sum regression. We find a significant association between the exposure mixture index all investigated health outcomes. Factors identified as risk factors include working in an enclosed heavily contaminated area, construction occupation, and exposure to blood and body fluids. Conversely, full-time employment emerged as a protective factor. This exposomics study emphasizes the importance of considering combined exposures. In an era marked by more frequent and severe natural disasters due to the evolving climate crisis, the exposomic framework holds promise as a valuable tool for disaster preparedness.

## Introduction

The World Trade Center (WTC) rescue and recovery responders were exposed to a complex mixture of smoke, dust, and debris generated by the collapse and long-lasting fires of the WTC buildings. In addition to exposure to chemicals, responders were exposed to traumatic psychosocial stressors, including fear for personal safety, injury, or illness, and working long hours and performing arduous work in chaotic conditions^1^. These exposures have been linked to increased risk for adverse health outcomes, including respiratory^2–5^, metabolic^6^, neurologic^7^, and mental health disorders^8–11^ Despite clear associations between responders’ experience and health outcomes, no studies have comprehensively examined the *overall* effect of the mixture of exposures that may have increased the risk for disease or promoted resilience against developing disease. Here, we apply an exposomics approach, combining chemical and non-chemical exposures with baseline (post-9/11) health and sociodemographic status, to encompass the variety of responders’ experiences during the rescue and recovery efforts. We refer to this concept as the *WTC exposome*.

Although several studies have attempted to identify risk factors associated with the onset and development of WTC-related diseases, most focus on individual factors selected a priori and use traditional regression approaches to examine associations between those individual factors (e.g., when started on the effort, work duration, previous respiratory symptoms, previous mental health symptoms, sociodemographic characteristics) and health outcomes^12–17^. However, at the time of 9/11 and in the months following the attacks, WTC responders were simultaneously exposed to a varying mixture of factors, rather than to one factor at a time. A growing body of literature suggests that co-exposure may influence the toxicity of individual factors. Under joint exposure, different factors may interact to cause synergistic or antagonistic effects^18^. One approach proposed to better capture responders’ exposure was to group responders into exposure categories. For example, Wisnivesky *et al*. (2011) categorized exposure into very high, high, intermediate, and low^16^ groups, based on three self-reported experiences (total time at the site, being caught in the dust cloud, and work on the pile arising from the collapse of the towers). While this approach helped link WTC exposure with increased risk of disease outcomes, it could not (nor claimed to) identify specific factors. As such, it does not provide information on the mixture’s overall impact or individual factors within the mixture that may increase or decrease the risk for adverse health effects.

The exposome, defined by C. Wild in 2005, is the totality of individuals’ exposure experience from conception until death and has been proposed to be critical for disease etiology^19^. The exposome can include toxicants, nutrients, drugs, microbiome metabolites, physical and psychosocial stress, lifestyle choices, and socioeconomic status. The exposome concept offers a data-driven approach to investigating the environmental causes of disease and can indicate a unique exposure profile rendering individuals more or less susceptible to the effects of stressors in their environment. Critical to this concept is that an individual’s “exposome” contains both risk and protective factors. In the context of the WTC responders, understanding the health effects of different combinations of exposures is important for identifying at-risk responders, as well as protective factors that may guide the development of appropriate interventions.

In this study, we adopt an exposomic-based, data-driven approach to systematically examine associations between WTC responders’ experience on 9/11 and during the rescue and recovery effort, and the risk of adverse health outcomes in 34,096 responders enrolled in the CDC/NIOSH World Trade Center Health Program General Responder Cohort (GRC). We define the “WTC exposome” to be the mixture of WTC-related experience, mental and physical health status, current/prior occupation, traumatic and environmental exposures, socioeconomic status, and social support during the work on the rescue and recovery effort. We hypothesize that different WTC exposomic profiles would be associated with the risk and resilience for five health outcomes (i.e., post-traumatic stress disorder; PTSD, gastroesophageal reflux disease; GERD, respiratory conditions, diabetes, and headaches) and examine how the mixture factors experienced by the Responders relate to their long-term health.

## Results

### Demographics

Sociodemographic characteristics are reported in Table 1. Participants’ mean age was 38 years on 9/11/2001 and 45 years at the time of their first WTCHP visit. Participants included in this study are primarily male (86.6%), White (56.3%), and non-Hispanic (76.6%). College Degree education was most common (63.8%), followed by High school or equivalent (20.8%).

### WTC-related health conditions

**Figure 1:**
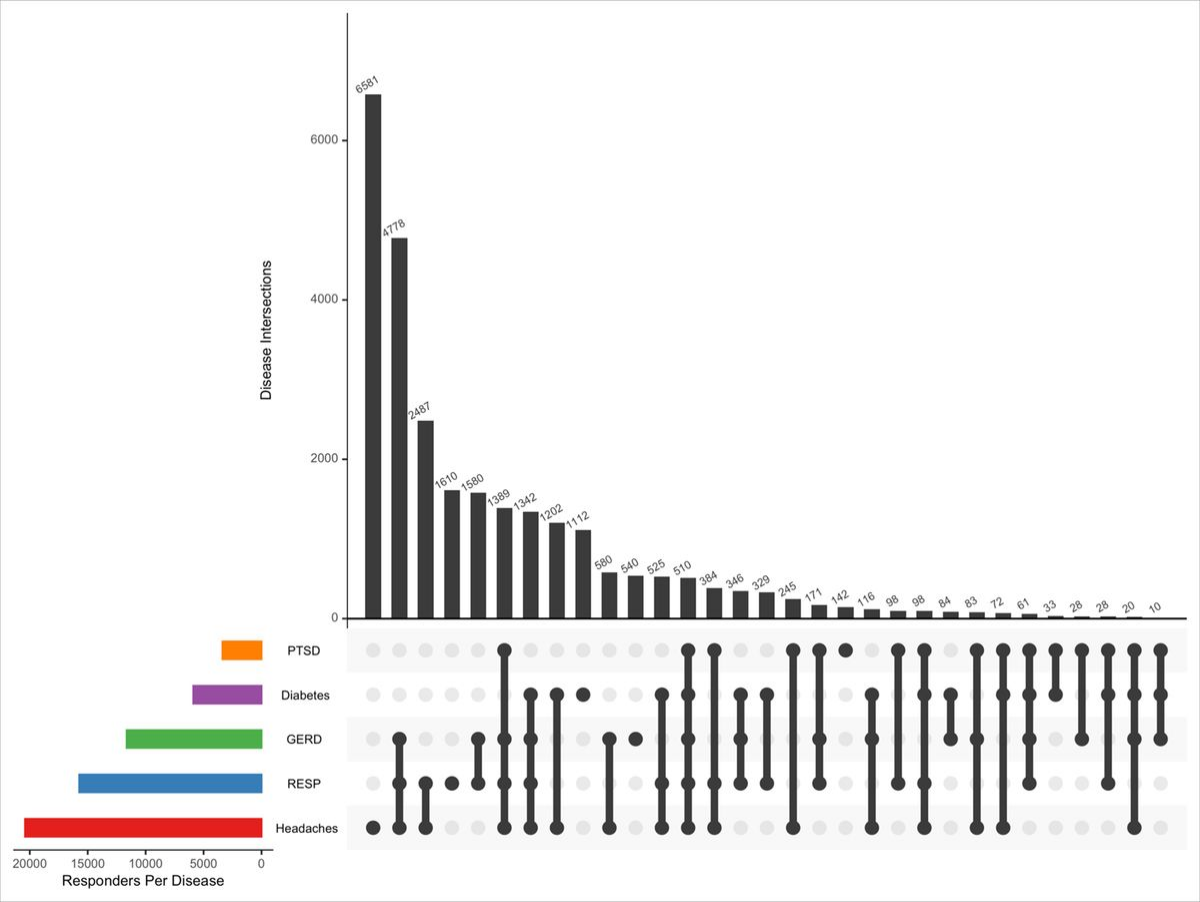


Frequencies of WTC-related physical or mental health conditions investigated in this study are reported in Table 2. Among the 34,096 responders included in this study, the most common health conditions were headaches (60%), respiratory disease (RESP) (46%), and gastroesophageal reflux disease (GERD) (34%). Diabetes was diagnosed in 17% of participants, and post-traumatic stress disorder (PTSD) was diagnosed in 10%. The co-occurrence of WTC-related health conditions is reported in Fig. 1. The most common health condition is headaches alone (n = 6,581). The most common subset of health conditions is the combination of GERD, RESP, and headaches (n = 4,778). The next most common combination is RESP and headaches (n = 2,487), followed by RESP alone (n = 1,610).

### World Trade Center Exposome and related health outcomes

Results from gWQS analysis show a significant association between the WTC-exposome and all investigated health outcomes in both the positive direction (i.e., factors associated with an increased likelihood of health outcomes; risk factors) and the negative direction (i.e., factors associated with a decreased likelihood of health outcomes; protective factors). Estimates, 95% confidence intervals, and p-values for each model are reported in Table 3.

### Risk and protective factors

**Figure 2:**
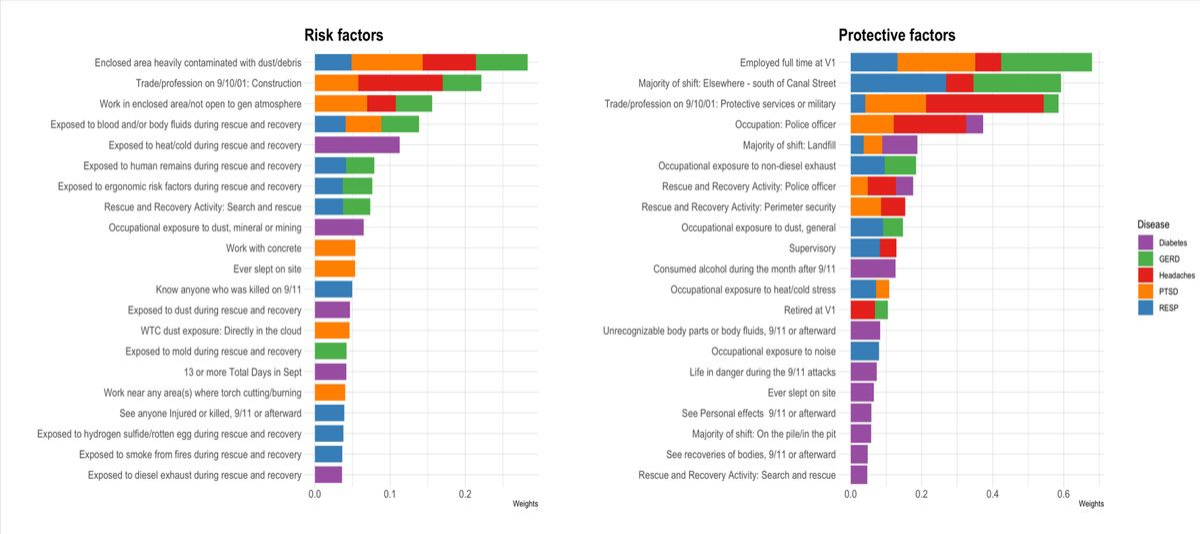


Weights from gWQS models are shown in Fig. 2. The main risk factors for WTC-related health conditions include working in an enclosed area heavily contaminated with dust/debris, construction occupation before 9/11/01, working in an area/not open to the general atmosphere, exposure to blood and body fluids, heat/cold, human remains, and ergonomic risk factors during rescue and recovery efforts, performing search and rescue activities, and occupational exposure to dust, mineral or mining. The main protective factors for WTC-related health conditions include being employed full-time at the first WTCHP visit, performing the majority of shift south of Canal Street but neither on the pile nor in the pit; protective services or military occupation before 9/11/01, and police officer occupation on 9/11.

For PTSD, working in an enclosed area heavily contaminated with dust/debris, working in an area not open to the general atmosphere, construction occupation before 9/11/01, working with concrete, and sleeping on site contributed most to the harmful effect of the WTC-exposome. In contrast, being employed full-time at the first WTCHP visit, protective services or military occupation before 9/11/01, and police officer occupation at 9/11 contributed most to the protective effect of the WTC-exposome. For GERD, working in an enclosed area heavily contaminated with dust/debris, construction occupation before 9/11/01, and being exposed to blood and body fluids during rescue and recovery efforts contributed most to the harmful effect of the WTC-exposome. In contrast, WTC responders who reported full-time employment at the first WTCHP visit and performing the majority of the shift elsewhere (south of Canal Street) experienced less GERD. For respiratory diseases, knowing anyone who was killed on 9/11, working in an enclosed area heavily contaminated with dust/debris, and being exposed to human remains during rescue and recovery contributed most to the harmful effect of the WTC-exposome. Performing the majority of shift elsewhere (south of Canal Street), and being employed full-time at the first WTCHP visit contributed most to the protective effect of the WTC-exposome on respiratory outcomes. For diabetes, being exposed to heat/cold during rescue and recovery efforts, occupational exposure to dust, mineral, or mining, and being exposed to dust during rescue and recovery working contributed most to the harmful effect of the WTC-exposome, while reporting to consumed alcohol during the month after 9/11, and performing the majority of shift at landfill contributed most to the protective effect of the WTC-exposome. For headaches, construction occupation before 9/11/01, working in an enclosed area heavily contaminated with dust/debris, and working in an area/not open to the gen atmosphere contributed most to the harmful effect of the WTC-exposome, while protective services or military occupation before 9/11/01, and police officer occupation at 9/11 contributed most to the protective effect of the WTC-exposome. Detailed exposure profiles as they relate to increased and decreased associations for each WTC-related health outcome is shown in Supplementary Fig. 1.

## Discussion

In this study, we applied an exposomic approach to systematically examine associations between World Trade Center (WTC) Responders’ experience and selected health outcomes. By considering all recorded exposures, both chemical and non-chemical, along with baseline (post-9/11) health and sociodemographic status, we captured the complexity of the WTC exposome and its effect on the long-term health of responders. Our findings demonstrate significant overall mixture effect of the WTC exposome on investigated health outcomes (PTSD, GERD, respiratory conditions, diabetes, and headaches), as well as the identification of risk and protective factors associated with the likelihood of adverse health outcomes.

Our exposomic approach revealed specific factors that contributed most to the harmful effects of the WTC exposome on health. The most prominent risk factor identified was working in enclosed areas heavily contaminated with dust and debris. This finding is consistent with prior research indicating that inhalation of the dust from the collapses during the rescue and recovery operations is the foremost cause of respiratory injuries^17,20^ and gastroesophageal reflux disease (GERD)^3^ in responders, and was also found to be associated with chronic PTSD symptoms^21^. Interestingly, factors associated with the total duration or frequency of work (i.e. - Number of days per month), being caught in the dust cloud, month work started on the effort, majority of the shift spent on the pile, all exhibited a comparatively modest impact on the overall outcome. Prior occupational exposure to dust, noise, diesel exhaust, heat/cold, gasoline, and welding fume was not determined to significantly contribute to the overall effect, highlighting the notion that mere occupational involvement in hazardous conditions may not hold the most significance in driving adverse health outcomes. Instead, they emphasize the critical importance of equipping responders with appropriate respiratory protection and prompting its utilization during disaster response efforts.

Other salient risk factors that have emerged pertain to psychological trauma experienced during the rescue and recovery, encompassing exposure to blood, bodily fluids, and human remains. These findings highlight the importance of preserving and strengthening the mental health support and resources extended to responders by the WTCHP. This encompasses early intervention initiatives, comprehensive support programs, routine mental health screenings, as well as the implementation of employer policies designed to foster overall resilience.

Importantly, our approach also identified factors within the WTC exposome that exhibited a protective effect against adverse health outcomes. Among these factors, being employed full-time at the first visit to the WTCHP and not working on the pile nor in the pit, were associated with a reduced likelihood of developing health conditions. These findings may be related to a “Healthy worker” effect, a phenomenon observed in occupational health studies where employed individuals tend to exhibit better health outcomes^22^. This effect occurs because people who are healthier and more physically able are more likely to remain in the workforce, while those with health issues may exit the workforce. Other identified factors associated with a decreased likelihood of disease, specifically those related to neurological health (PTSD and headaches), include being a police officer or military. These findings may be related to prior training and exposure to similar stressors. Another explanation is that the protective effect we detect may result from report bias due to underreported mental health concerns among police and military personnel. Rates of mental health reports remain disproportionately low within these populations due to fear of professional consequences and stigmas surrounding mental health^17^.

The use of an exposomics approach in this study has several strengths. By considering a wide range of exposures and their interactions, the study provides a more complete understanding of the complex mixture of factors that responders were exposed to during the WTC rescue and recovery efforts. The weighted quantile sum (WQS) regression analysis identified harmful and protective factors within the WTC exposome, providing valuable insights for targeted interventions and preventive measures. When interpreting the results of this study, some limitations should be considered. The study relied on self-reported information and retrospective data, which may be subject to recall bias. Additionally, while the gWQS regression analysis accounted for potential confounders and estimated the association between the weighted index and health outcomes, causality cannot be established due to the study’s observational nature. Further research, including leveraging longitudinal data collected under the WTCHP, is needed to confirm these findings.

In conclusion, this study contributes to our understanding of the health effects of the WTC exposome on responders and may guide policy and regulatory decision-making regarding responders’ health preparedness in the event of future disasters. By adopting an exposomics approach, the study provides valuable insights into the specific exposome factors associated with an increased or decreased risk of adverse health outcomes. These findings may inform the development of targeted interventions and support the well-being of WTC responders.

## Methods

### Study design and participants

This study includes 34,096 enrollees of the World Trade Center Health Program (WTCHP) General Responder Cohort (GRC) who 1) consented to have their data aggregated for research purposes, 2) had an Exposure assessment questionnaire (EAQ) record, and 3) had mental health diagnostic interview. The GRC has been described in detail by Dasaro *et al*. 2017^23^. Briefly, participants were monitored at any of the seven CDC/NIOSH -funded Clinical Centers of Excellence (CCE) and completed an initial ‘baseline’ visit. All members worked or volunteered on WTC rescue and recovery efforts following the 9/11 attack on the WTC towers, meeting at least one of the following criteria: (i) involved for ≥ 4 hours from September 11 to September 14, 2001, or ≥ 24 hours in September 2001, or ≥ 80 hours from September 11 to July 2002; (ii) member of the Office of the Chief Medical Examiner for New York City, handling and processing human remains for ≥ 4 hours; (iii) worked for the Port Authority Trans Hudson Corporation (PATH), cleaning PATH tunnels for ≥ 24 hours between February 2002 and July 2002. At the baseline visit, responders completed self- and interviewer-administered questionnaires. These instruments collected information on sociodemographic characteristics, exposure history, medical conditions, and mental health symptoms^23^. Physical examinations, clinical chemistry laboratory tests, and pulmonary function tests (PFTs) were also performed. Questionnaires used for this study are summarized in supplementary table 1.

### WTC-Related Health Outcomes

For our outcomes, we selected WTC-certified health conditions and non-certifiable conditions. The WTCHP uses a certification mechanism to provide medically necessary treatment to responders. WTC health conditions are determined by the designated CCE or National Provider Network (NPN) physician during the baseline health evaluation, a monitoring visit, or a treatment visit^24^. To diagnose a WTCHP member with a health condition, evidence collected from the member’s visits is used to justify certification of a condition. A combination of medical history, physical examination, present symptoms, exposure assessment and other questionnaires (summarized in supplementary table 1), and diagnostic testing (e.g., PFTs, imaging, chemical laboratory tests) are used to arrive at diagnoses. The Diagnostic Interview Schedule (DIS) is administered to every responder at the second visit and assesses psychological trauma, PTSD, depression and alcohol consumption. The temporal relationship between the onset of symptoms of a condition and WTC exposures is also considered^25^. For the program to certify a condition, the following criteria need to be met: (i) the health condition is included on the List of WTC-Related Health Conditions; (ii) exposures present during WTC rescue and recovery efforts play a significant role in aggravating, causing or contributing to the physical and mental condition exhibited by the responder during their clinical evaluation. Certified conditions are categorized into the care suites: acute traumatic injuries, airway and digestive disorders, cancers, mental health conditions, and musculoskeletal disorders. Responders certified for any condition within a care suite do not need further certification to receive treatment for other conditions within the same care suite.^4^ Non-certified conditions are WTC-related health problems that do not meet the criteria described above. Some of these outcomes can be described as potential WTC-related diseases as research about their associations with WTC exposures is ongoing. Examples of these potential conditions include late-onset diabetes and headaches^6,23^.

### Statistical analyses

#### Data preparation

From 239 variables gathered from the baseline surveillance dataset, we generated univariate summary statistics and examined distributions. To standardize all exposure data, we re-coded each of the variables as a binary indicator (0/1) where zero indicated not present and one indicated present. Only items occurring in 10–90% of responders were included in further analyses, resulting in the selection of 84 factors. Covariates that are known predictors of health or strong potential confounders included a priori in the models, based on biological plausibility, were gender, age at the time of 9/11, and race.

#### Generalized weighted quantile sum (gWQS) regression

To comprehensively investigate how the WTC baseline exposome is associated with each health outcome (PTSD, GERD, respiratory problems, diabetes, and headaches), we used generalized weighted quantile sum (gWQS) regression. The gWQS has been described in detail in Carrico *et al*. 2015^26^. In brief, the gWQS is a mixtures-based ensemble modeling strategy that tests for associations between the combined effect of multiple exposures and an outcome of interest. It estimates a weighted index of all factors and performs inference on the regression coefficient that characterizes the association between the outcome and this weighted index. gWQS randomly splits the initial dataset into training and validation sets (i.e., 40% vs. 60%). In the training set, gWQS constructs a weighted additive index (∑_j_ = 1[w_j_q_ij_]) of all factors previously ranked in quantiles (qij). To facilitate the implementation and interpretation of the gWQS approach, all factors are harmonized to have a standard scale before ranking them. Each weight (wj) is mapped to an individual factor (j) of the mixture, and the mixture is constrained to be positive or negative. Additionally, all weights are constrained to sum to one, enabling sorting by relative importance. Factors that impact the association between the mixture index and the outcome have larger weights; factors with little or no impact have near-zero weights. To reduce the high correlation between factors and have more accurate and variable weight estimates, gWQS employs a bootstrap approach, randomly selecting samples from the training set. Here, a total of 10,000 bootstrap samples were generated from the training data to estimate the WQS index weights, and the effect of the index was assessed for statistical significance in the validation set. Thus, the test for the significance of β_1_ was based on independent data.

WQS models were implemented in R (v4.0.2) with the gWQS package.

## Figures and Tables

**Table 1:** Sociodemographic characteristics of participants included in this study.

	WTCHP-GRC participants (N=34096)

**Age at 9/11/2001 (years)**	38.0 [13.0, 78.0]
**Age at First Visit (years)**	45.0 [20.0, 89.0]
**Gender**	
Female	4566 (13.4%)
Male	29487 (86.6%)
**Race**	
American Indian and Alaskan Native	86 (0.253%)
Asian	390 (1.15%)
Black or African American	3126 (9.18%)
Multi-Racial	3845 (11.3%)
Pacific Islander	38 (0.112%)
White or Caucasian	19186 (56.3%)
Unknown	7382 (21.7%)
**Ethnicity**	
Hispanic	6179 (23.4%)
Non-Hispanic	20262 (76.6%)
**Education**	
Less than high school diploma	2253 (6.92%)
High school or equivalent	6764 (20.8%)
College Degree (or some college)	20747 (63.8%)
Graduate Degree	2780 (8.54%)

**Table 2.** Frequencies of WTC-related physical or mental health conditions investigated in this study (N = 34, 096)

	Overall (N=34096)

**PTSD**	
No	30724 (90.1%)
Yes	3372 (9.89%)
**Diabetes**	
No	28208 (82.7%)
Yes	5888 (17.3%)
**GERD**	
No	22458 (65.9%)
Yes	11638 (34.1%)
**Respiratory**	
No	18360 (53.8%)
Yes	15736 (46.2%)
**Headaches**	
No	13684 (40.1%)
Yes	20412 (59.9%)

**Note** PTSD: post-traumatic stress disorder, GERD: gastroesophageal reflux disease

**Table 3.** Results from gWQS analyses among 34,096 WTC responders included in this study. For each health condition, associations between the WTC exposome and outcomes were modeled in the positive (risk factors) and the negative (protective factors) directions.

Outcome	Direction	Estimate	CI (95%)	P-value

PTSD	Risk factors	6.4	4.8–8.4	< 2e-16 ***
	
	Protective factors	0.13	0.11–0.17	< 2e-16 ***
	
Diabetes	Risk factors	1.57	1.25–1.97	0.00011***
	
	Protective factors	0.71	0.59–0.85	0.00014***
	
GERD	Risk factors	4.36	3.66–5.19	< 2e-16 ***
	
	Protective factors	0.46	0.39–0.54	< 2e-16 ***
	
RESP	Risk factors	3.86	3.32–4.49	< 2e-16 ***
	
	Protective factors	0.59	0.51–0.70	1.21e-10 ***
	
Headaches	Risk factors	5.57	4.68–6.64	< 2e-16 ***
	
	Protective factors	0.32	0.29–0.36	< 2e-16 ***

CI: confidence interval, PTSD: post-traumatic stress disorder, GERD: gastroesophageal reflux disease, RESP: Respiratory disease

## Data Availability

Anonymised data and supporting documents are available upon request from the General Responder Data Center of the WTCHP via christopher.dasaro@mssm.edu. A Data Transfer and Use Agreement and IRB approval is required before data release.
